# Fault Detection for Wind Turbine Blade Bolts Based on GSG Combined with CS-LightGBM

**DOI:** 10.3390/s22186763

**Published:** 2022-09-07

**Authors:** Mingzhu Tang, Caihua Meng, Huawei Wu, Hongqiu Zhu, Jiabiao Yi, Jun Tang, Yifan Wang

**Affiliations:** 1 School of Energy and Power Engineering, Changsha University of Science & Technology, Changsha 410114, China; 2 Hubei Key Laboratory of Power System Design and Test for Electrical Vehicle, Hubei University of Arts and Science, Xiangyang 441053, China; 3 School of Automation, Central South University, Changsha 410083, China

**Keywords:** wind turbine, SMOTE, class imbalance, Gaussian mixture model, cost-sensitive, LightGBM

## Abstract

Aiming at the problem of class imbalance in the wind turbine blade bolts operation-monitoring dataset, a fault detection method for wind turbine blade bolts based on Gaussian Mixture Model–Synthetic Minority Oversampling Technique–Gaussian Mixture Model (GSG) combined with Cost-Sensitive LightGBM (CS-LightGBM) was proposed. Since it is difficult to obtain the fault samples of blade bolts, the GSG oversampling method was constructed to increase the fault samples in the blade bolt dataset. The method obtains the optimal number of clusters through the BIC criterion, and uses the GMM based on the optimal number of clusters to optimally cluster the fault samples in the blade bolt dataset. According to the density distribution of fault samples in inter-clusters, we synthesized new fault samples using SMOTE in an intra-cluster. This retains the distribution characteristics of the original fault class samples. Then, we used the GMM with the same initial cluster center to cluster the fault class samples that were added to new samples, and removed the synthetic fault class samples that were not clustered into the corresponding clusters. Finally, the synthetic data training set was used to train the CS-LightGBM fault detection model. Additionally, the hyperparameters of CS-LightGBM were optimized by the Bayesian optimization algorithm to obtain the optimal CS-LightGBM fault detection model. The experimental results show that compared with six models including SMOTE-LightGBM, CS-LightGBM, K-means-SMOTE-LightGBM, etc., the proposed fault detection model is superior to the other comparison methods in the false alarm rate, missing alarm rate and F1-score index. The method can well realize the fault detection of large wind turbine blade bolts.

## 1. Introduction

As a leader of global wind energy development, China’s installed wind power capacity ranked first in the world by 2021. Additionally, in light of China’s double carbon reduction targets, it is essential to address the fault issue with the earlier wind turbine installations in addition to increasing the overall installed capacity of wind turbines. Therefore, the fault detection of wind turbines is particularly important in the background of China’s double carbon goals. Simultaneously, it also promotes the development of wind turbine fault detection technology [1]. At present, in the field of wind turbine fault detection, the fault detection of wind turbine impeller blades has become a research focus. As the main component of the wind turbine [2], the wind turbine impeller blades operate in harsh environments and are subjected to alternating loads for a long time, which leads to a number of failure issues. For example, when the impeller rotates, it is easy to cause the loosening fault of the blade bolt under the external alternating load, and can even lead to the fracture fault of the bolt in serious cases [3]. The failure of blade bolts directly affects the aerodynamic performance of the impeller, which causes additional load and load imbalance of the wind turbine, and finally reduces the overall life of the wind turbine. Therefore, it is vitally necessary to study the fault detection method of wind turbine blade bolts. At the same time, it is of great significance in reducing the maintenance cost of the wind turbine and increasing the power generation of the wind turbine.

The fault detection methods of blade bolts mainly include vibration signal detection technology [4], the finite element analysis method [5], and the machine learning method [6]. In recent years, machine learning has been widely used in the field of bolt failure detection. Reference [7] used the damage index of multi-scale fuzzy entropy to extract the important features of bolts to construct a dataset, and then input it into the least squares support vector machine classifier to detect the fault of bolts. Reference [8] performed visual processing and extracted relevant features on the collected bolts’ image information to realize the bolt-loosening detection, and the feasibility of the proposed method was verified in the simulation experiment. Reference [9] combined deep learning and the mechanical-based manual-torque method for bolts’ fault detection, which effectively reduced the cost of artificial detection and improved the performance of fault detection models.

When the machine-learning method is used to detect the fault of wind turbine components, processing the data class imbalance problem has also become a hotspot in the research. In the field of machine learning, common methods for processing data class imbalance include oversampling, undersampling, cost-sensitive learning, and optimized classification algorithms [10]. The main research method involved in this paper is the oversampling method. The representative methods of oversampling include random oversampling [11], SMOTE (Synthetic Minority Oversampling technique) [12], Borderline-SMOTE [13], K-means SMOTE [14], etc. Since the samples expanded by random oversampling originate from the raw samples, it is less productive for the training and learning of the model, so domestic and foreign researchers mostly use SMOTE to simply expand the fault samples.

Obtaining high-quality samples is susceptible to noise samples and distribution characteristics of raw data. The direct use of SMOTE to expand data samples can achieve a certain effect, but it is easy to cause data redundancy problems for datasets with extreme data class imbalance. Improved variants of SMOTE are generally used to obtain high-quality synthetic samples. For example, Li et al. [15] proposed a hybrid cluster–boundary fuzzification sampling method to solve the problem of low accuracy of detection models caused by a data class imbalance, which not only balanced data class but also alleviated data redundancy issues. However, this method does not completely consider the original distribution characteristics of the fault data. Aiming at the intrusion detection data class imbalance problem, Zhang et al. [16] combined traditional SMOTE and random undersampling based on GMM to achieve the purpose of balancing the dataset. Yi et al. [17] proposed a SMOTE sampling method based on fault class clustering to solve the problem of data class imbalance in SCADA of wind turbines, which solved the problem that traditional SMOTE does not consider the distribution characteristics of the original fault samples when synthesizing samples. However, the above methods are susceptible to noise samples.

Cost-sensitive learning is also widely used to process the problem of data class imbalance. Aiming at the problem of class imbalance in the operational data of wind turbine gearboxes, Tang et al. [18] introduced a cost-sensitive matrix into the LightGBM algorithm, which solved the problem of inferior performance of the detection model due to the data class imbalance. In order to make the cost-sensitive learning independent of the manually designed cost-sensitive matrix, Reference [19] proposed an innovative method based on the genetic algorithm program to construct the cost-sensitive matrix independent of the manual design. Then, the improved cost-sensitive matrix was used to adjust the attention weight of the correlation classification algorithm to the fault class samples. The method of introducing cost-sensitive learning into the classification algorithm has been well verified when solving the problem of data class imbalance in various fields. However, when the class imbalance problem of the dataset is serious, only using cost-sensitive learning to adjust the learning weight of the classifier on the fault class samples, the effect is not so obvious.

LightGBM is widely used in the field of wind turbine fault detection due to its strong classification performance and fast training speed [20]. For example, aiming at the problem of frequent faults of wind turbine gearboxes. Tang et al. [21] proposed a fault diagnosis model for wind turbine gearboxes based on adaptive LightGBM, which was well verified in practical cases. Wang et al. [22] constructed a cascaded SAE abnormal state detection and LightGBM abnormal state classification model detection framework, which solved the problem that the wind turbine alarm system is insensitive to early faults. However, when using the fault detection model based on LightGBM for wind turbine fault detection, due to the problem of data class imbalance, its fault detection performance will be difficult to guarantee.

Therefore, aimed at the above problems, this paper proposes a fault detection method for wind turbine blade bolts based on GSG combined with CS-LightGBM. This method combined the new sampling method proposed in this paper with cost-sensitive learning, and then solved the problem of class imbalance in the bolt operation data of wind turbine blades.

## 2. Materials and Methods

### 2.1. Wind Turbine Blade Bolts Failure Problem Description

Wind turbine blades are the main components of wind turbines to capture wind energy [23]. Common wind turbine impellers are generally 3-blade type, which are connected by the hub. The blade converts the kinetic energy carried by the flowing wind into its own rotational kinetic energy to drive the hub to rotate at the same time, and then the hub transfers the kinetic energy to the main shaft of wind turbines [24]. The rotational speed of the main shaft of the non-direct-drive wind turbine is synchronized with the rotational speed of the hub, which requires a gearbox to increase the lower rotational speed from the main shaft to the rated rotational speed of the wind turbine [25]. Through the above analysis of the energy transfer process, once the wind turbine blade has a fault in operation, it will affect the entire power generation process of the wind turbine. The wind turbine blade bolt connects the blade to the hub. Once the blade bolt fails, such as bolt loosening or bolt breakage, it will directly affect the aerodynamic balance performance of the entire impeller. The aerodynamic imbalance of the wind turbine impeller directly affects the operation of the gearbox, so the aerodynamic imbalance of the wind turbine impeller is also one of the reasons for the failure of the gearbox [26]. In addition, it will also cause vibration of the wind turbine body. Therefore, it is very important to detect the fault of wind turbine blade bolts.

**Figure 1:**
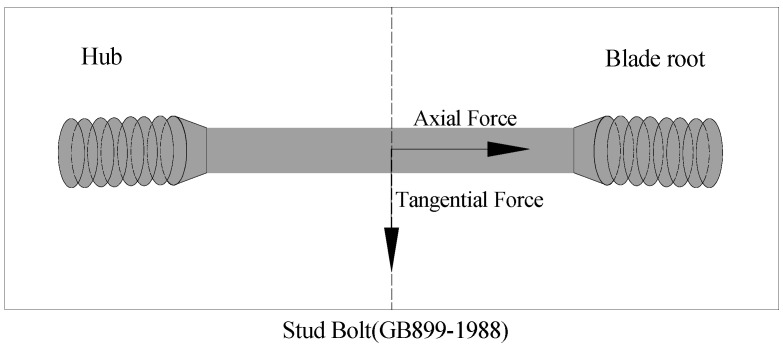
Force Analysis of Blade Bolts.

Wind turbine blade bolts are generally stud bolts [27]. Take the bolt of type GB899-1988 as an example, as shown in Figure 1; one end is connected to the hub, and the other end is fastened at the root of the blade by pre-embedding. The main dimensional parameters of the wind turbine stud bolts are as follows: nominal diameter: 470 mm, outer diameter: 30 mm, pitch of screw: 2 mm, and screw diameter: 364 mm. When the blade rotates, the blade bolt is mainly subjected to an axial force and a tangential force. Under the combined action of axial force and tangential force, the bolt will be subjected to an alternating load, resulting in fatigue breaking of bolts. However, the weak alternating load generally does not cause the bolt fault problem, which generally occurs when the bolt is subjected to strong load impact. For example, when the wind turbine during operation encounters wind shear, emergency shutdown, etc., it is easy to cause the blade to be impacted by a strong load, causing the blade bolt to fail. In addition, the cause of its failure is also related to the quality of the steel product of the bolt [28,29].

**Figure 2:**
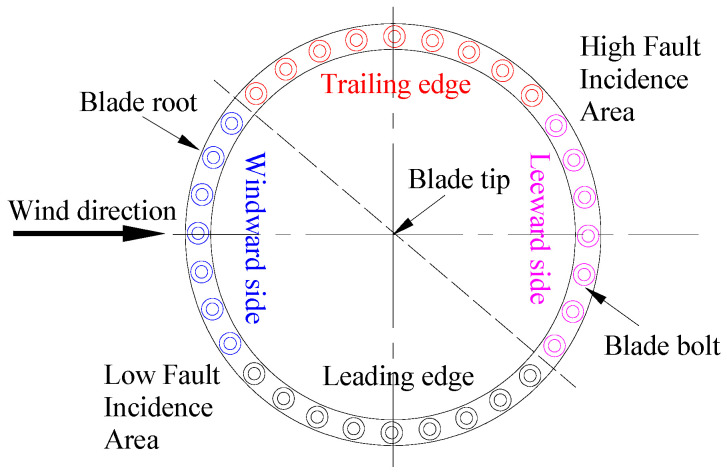
Failure distribution of bolts at different positions.

The failure rate of the blade bolts is closely related to the distribution of the bolts at the blade root. Since the force that causes the failure of the blade bolt is mainly the tangential force, the failure rate of the bolt that is subjected to the tangential force for a long time is relatively high. Based on the working principle of wind turbine blades and field experience, the failure rates of bolts at different positions of the blade root were obtained, as shown in Figure 2. In Figure 2, the blade tip is vertically outward, and the dotted line is used as the dividing line. Above the dotted line represents the high-incidence area of faults; below the dotted line represents the low-incidence area of faults. Therefore, the bolt failure rate at the trailing edge and leeward side of the blade is higher than that at other locations. When collecting bolts’ operation-monitoring data, the data sensors are mostly located at the bolt position on the trailing edge and leeward side of the blade. The advantage is that the collected data can provide more useful information to the machine-learning algorithm [30].

**Figure 3:**
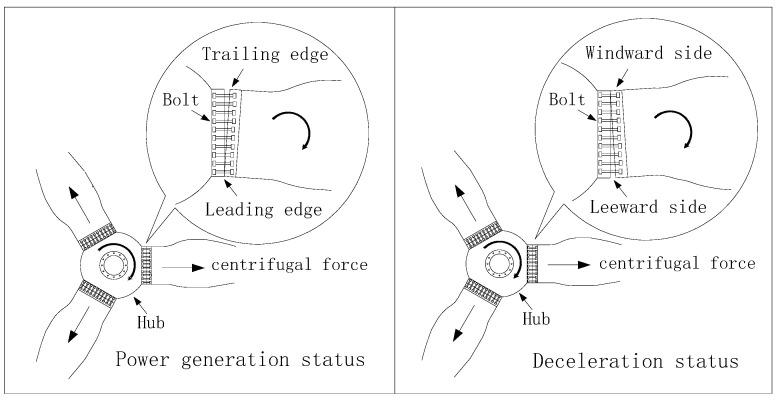
The state of the blades of the wind turbine in different operating states.

The common failures of wind turbine blade bolts are mainly bolt loosening and bolt breakage. Figure 3 shows the state of the blades of the wind turbine in different operating states. As shown in Figure 3, no matter whether the wind turbine impeller is in the power generation status or the deceleration status, the blade bolt will be subjected to a centrifugal force [31]. However, according to the mechanical analysis, the main reason for the failure of blade bolts is not the centrifugal force but the tangential force. Bolts situated the trailing edge of blades are subjected to a tangential force when wind turbines operate in power generation status. When the wind turbine impeller is in a state of deceleration, especially when the wind turbine is emergency shutdown, the bolt on the leeward side of the blade is also subjected to a tangential force. Therefore, the repeated action of the alternating load leads to the failure problem of the wind turbine blade bolt.

Through the above analysis, the problem of wind turbine blade bolt fault detection is regarded as a binary classification problem. However, for a wind turbine bolts dataset, there is a class imbalance problem because the samples of the fault class are far fewer than the samples of the normal class. As a result, the failure detection performance of wind turbine blade bolts is lower than the required detection accuracy. Aiming at the problem, a fault detection method for wind turbine blade bolts based on GSG combined with CS-LightGBM is proposed.

### 2.2. GSG Oversampling Method

#### 2.2.1. GMM

The Gaussian Mixture Model (GMM) is often used for sample clustering, and its principle is based on a Gaussian distribution model. Multiple single Gaussian models are integrated to obtain a GMM [32]. When the GMM is used to process data samples, theoretically, as long as the number of integrated components is enough, it can fit the original distribution structure of any data sample as much as possible. Generally, GMM can be described from the perspective of geometric models or mixture models. Although the perspectives on describing models are different, the conclusions obtained are the same. In this paper, we describe the GMM in terms of a mixture model and input a dataset D= {x_1_, x_2_, x_3_, ..., x_N_} to the GMM. A hidden variable Z is introduced into dataset D for subsequent calculation, denoted as (D, Z) = {(x_1_, z_1_), (x_2_, z_2_), ..., (x_N_, z_N_)}. The probability density function of GMM is as Equation (1):

(1) $$P\left(x|\theta \right)=\overset{K}{\underset{k=1}{\sum }}P_{k}\cdot N\left({x|\mu }_{k},\Sigma_{k}\right)$$

where θ = {P_1_, ..., P_k_, μ_1_, ...., μ_k_, ∑_1_, ..., ∑_k_}, P_k_ represents the probability of the kth Gaussian distribution, μ_k_ represents the mean of the kth Gaussian distribution, *∑*_k_ represents the covariance matrix of the kth Gaussian distribution, x is the observation sample, K is the number of Gaussian distributions, and N is the number of samples. GMM uses MLE to estimate the optimal parameter θ_MLE_, as in Equation (2).

(2) $$\theta_{\mathrm{MLE}}=\arg\underset{\theta }{\max}\overset{N}{\underset{i=1}{\sum }}\log\overset{K}{\underset{k=1}{\sum }}P_{k}\cdot N\left({x|\mu }_{k},\Sigma_{k}\right)$$

In order to solve θ_MLE_, the θ parameter must be calculated first, so GMM uses the EM algorithm to calculate the θ parameter. The EM algorithm calculates the θ parameter through E steps and M steps. The purpose of the E step is to calculate the expected value of the logarithmic joint probability of (D, Z) under the posterior probability distribution, denoted as Q (θ, θ^t^), as in Equation (3).

(3) $$Q\left(\theta ,\theta^{t}\right)=\underset{Z_{1}\ldots Z_{N}}{\sum }\overset{N}{\underset{i=1}{\prod }}P\left(Z_{i}|x,\theta^{t}\right)\cdot \left[\mathrm{logP}\left(x_{1},Z_{1}|\theta \right)+\ldots +\mathrm{logP}\left(x_{N},Z_{N}|\theta \right)\right]$$

The purpose of the M step is to maximize the expected value of Q (θ, θ^t^), so as to obtain the best estimated parameters P_j_, μ_j_, ∑_j_ with the iteration, and define γ_ij_ as Equation (4).

(4) $$\gamma_{\mathrm{ij}}=\frac{P_{j}\cdot N\left(x_{i}|\mu_{j},\sum_{j}\right)}{\sum_{k=1}^{N}P_{k}\cdot N\left(x_{i}|\mu_{k},\sum_{k}\right)}$$

So, the three parameter update formulas of GMM are as follows:

(5) $$P_{j}^{t+1}=\frac{1}{N}\sum_{i=1}^{N}\gamma_{\mathrm{ij}}$$

(6) $$\mu_{j}^{t+1}=\frac{\sum_{i=1}^{N}\gamma_{\mathrm{ij}}x_{i}}{\sum_{i=1}^{N}\gamma_{\mathrm{ij}}}$$

(7) $$\sum_{j}^{t+1}=\frac{\sum_{i=1}^{N}{\left(x_{i}-\mu_{j}\right)}^{T}\left(x_{i}-\mu_{j}\right)\gamma_{\mathrm{ij}}}{\sum_{i=1}^{N}\gamma_{\mathrm{ij}}}$$

In order to determine the optimal number of components to obtain the optimal clustering, GMM generally uses the Bayesian information criterion (BIC) or Akaike information criterion (AIC) to determine the optimal number of components [33]. This paper uses BIC to determine the optimal number of components, which is defined as Equation (8):

(8) BIC=ln(n)k−2ln(L)

where k is the number of model parameters, n is the number of samples, and L is the likelihood function [34].

#### 2.2.2. SMOTE

**Figure 4:**
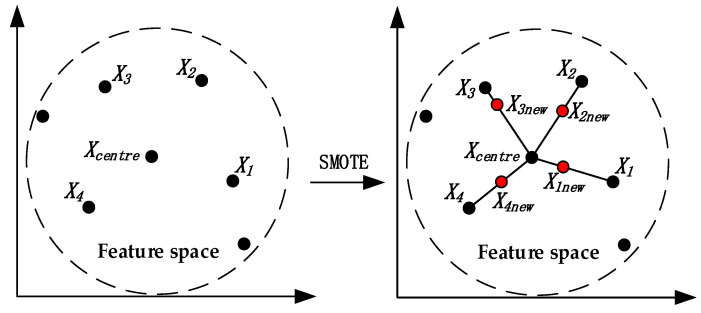
The sampling schematic of SMOTE (k = 4, sampling strategy = 1).

The main idea of the Synthetic Minority Oversampling Technique (SMOTE) is that the neighboring samples in the same feature space have a certain similarity [35]. Therefore, the artificial samples synthesized by neighboring samples also have a certain similarity with the original samples [36]. The traditional random oversampling method achieves the purpose of sample balance by randomly copying some original samples, but this method does not provide much useful information to the classifier. It even causes data redundancy and overfitting of the algorithm model. Since SMOTE does not simply copy the original data samples, it can avoid the above problems to a certain extent. Many practices have proved that SMOTE can effectively improve the performance of classifiers in binary and multi-classification problems. Figure 4 shows the sampling schematic for SMOTE; here, the number of majority class samples is 11. The specific sampling process is as follows:

1. Randomly select an initial sample in the minority class samples feature space as the nearest neighbor sample center X_centre_;
2. According to the principle of K nearest neighbors, take X_centre_ as the center to obtain k sample points, denoted as X_i_ (i = 1, 2, ..., k);
3. Set a sampling strategy based on the total number of expanded samples;
4. According to Formula (9), synthesize new samples X_inew_.

(9) $$X_{\mathrm{inew}}=X_{\mathrm{centre}}+\mathrm{rand}(0,1)\times (X_{i}-X_{\mathrm{centre}})$$

#### 2.2.3. GSG Sampling Method

When the wind turbine fails, its control system will immediately stop the equipment operation and remind maintenance personnel to fix the fault. The data collector is installed at the bolt of wind turbine blades, once the collected data have abnormal problems, even if it is only a small abnormal problem. In order to prevent the expansion of the abnormal problem, the measures taken by the maintenance personnel are to shut down the wind turbine and check the abnormal situation. The above measures result in only a small number of fault datasets in the wind turbine blade bolts’ dataset, which leads to a class imbalance problem in the dataset.

Aiming at the problem of data class imbalance, the common solution is to increase the number of minority class samples. Increasing minority class samples is generally achieved through SMOTE. However, it will lead to data redundancy problems. In addition, the traditional SMOTE does not consider the structural distribution and density distribution of samples within the class, and is easily affected by noise samples. Aiming at the above problems, this paper proposes an oversampling method based on GSG. This method does not just make the classes of the dataset balanced, but expands the minority class samples to a certain scale under the constraint of not changing their original distribution characteristics as much as possible. Thereby alleviating the class imbalance problem of the dataset.

**Figure 5:**
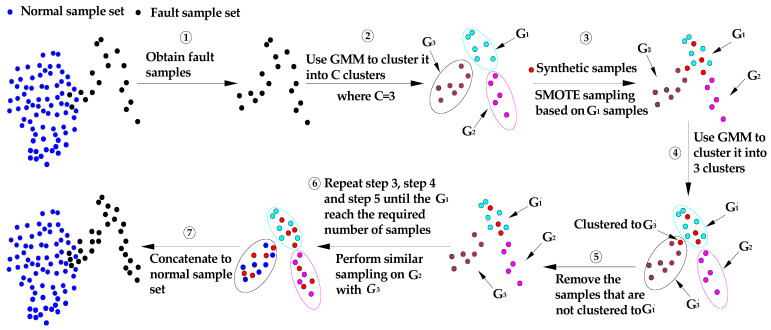
Basic principle of sampling based on GSG.

Figure 5 shows the basic principle of sampling based on GSG. The basic idea is to start from the fault sample set in the training set. Firstly, use the GMM based on the optimal number of components to cluster the fault sample set into C clusters, and obtain the samples of each cluster. Then, calculate the ratio of the number of samples in each cluster to the total number of fault samples, and multiply the ratio by the total number of samples to be expanded to obtain the number of subsamples to be expanded in each cluster. Finally, perform SMOTE oversampling based on samples of each cluster. This ensures that the original density distribution of the fault samples is not changed when using SMOTE sampling. Use the GMM with the same initial center of the cluster again to cluster the fault samples with added synthetic samples into C clusters. The synthetic samples that are not clustered into the corresponding clusters will be removed. Repeat the above process until each cluster has been expanded. This ensures that the structural distribution characteristics of fault samples can be preserved to the greatest extent when using SMOTE to synthesize samples.

**Figure 6:**
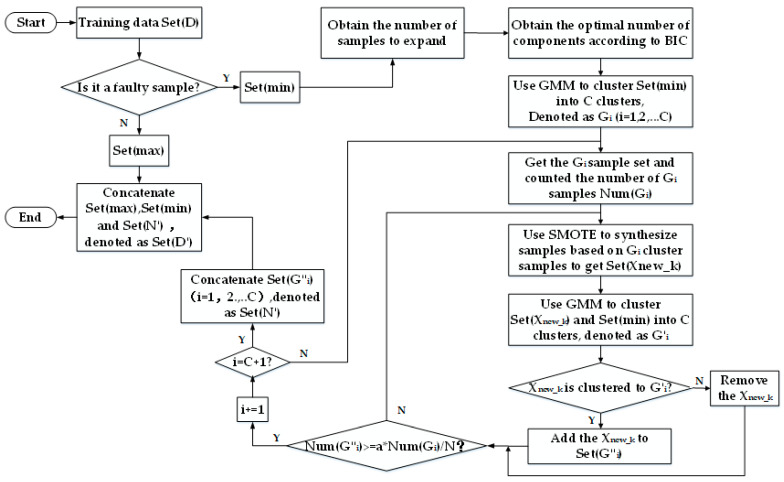
The oversampling Process Based on GSG.

Figure 6 shows the oversampling process based on GSG. Its specific description is as follows:

1. Perform data preprocessing. First, remove the missing values and abnormal values in the wind turbine blade bolts dataset, and then calculate the feature importance through XGboost and select the important features. Finally, divide the processed dataset is into a training set and validation set. In order to ensure the validity of the experiment, only the training set samples are oversampled. We denoted the training dataset of wind turbine blade bolts as Set(D).
2. Set(D) contains two types of data samples. One is the normal data sample with a large number of samples, denoted as Set(max) = {X’_1_, X’_2_, …, X’_M_}; the other is the fault data sample with a small number of samples, denoted as Set(min) = {X_1_, X_2_, …, X_N_}.
3. Based on the original dataset, calculate the class imbalance rate r (r = N/M), and generate a random floating point number b with an interval between (r, 1) as the sampling strategy. Then, obtain the number of samples to be expanded in Set(min), denoted as parameter a. (a = int (M*b − N), where M represents the number of samples of Set(max), and N represents the number of samples of Set(min)).
4. Train Set(min) according to BIC to obtain an optimal number of components: C. Use GMM to cluster Set(min) into C clusters. We denoted each cluster as G_i_ (i = 1, 2, ..., C).
5. Obtain G_i_ (i = 1, 2, ..., C) samples, and count the number of G_i_ samples, denoted as Num (G_i_), which is used to determine the needed number of synthesized samples in the G_i_ cluster, denoted as int (a*Num (G_i_)/N). This ensures that the sample density distribution of the original Set(min) tends to remain unchanged.
6. Use SMOTE to synthesize new samples based on the G_i_ cluster samples to obtain a new sample set, denoted as Set (X_new_k_) (k = 1, 2, ..., a*Num (G_i_)/N). Use GMM with the same initial center of the cluster to cluster the Set (X_new_k_) samples set and G_i_ cluster samples into C clusters again, denoting each cluster as G’_i_ (i = 1, 2, ..., C).
7. Create an empty dataset Set(G″_i_). If the X_new_k_ sample is clustered into the G′_i_ cluster, add the X_new_k_ sample to the dataset Set(G″_i_), and calculate the number of Set(G″_i_) samples, denoted as Num (G″_i_). Otherwise, remove the X_new_k_ sample. This ensures that the structural distribution characteristics of the original dataset Set(min) can be preserved to the greatest extent when synthesizing samples.
8. If Num (G″_i_) < a*Num (G_i_)/N, repeat steps (6) and (7); otherwise, repeat steps (5), (6) and (7), until each cluster is expanded.
9. Concatenate the dataset Set(G″_i_) (i = 1, 2, ..., C), denoted as Set(N′).
10. Concatenate Set(max), Set(min) and Set(N′). Then, return a training Set(D′) with more minority class samples than the original training Set(D).

This paper gave the pseudo-code of GSG, as shown in Algorithm 1:
**Algorithm 1** GSG Pseudo-code**Input:**  Training Set(D) = {Set(min), set(max)}  |Set(min)|=N,|Set(max)|=M # N is the number of fault class samples.                     # M is the number of normal class samples.**Output:**  A training Set (D′) with more fault class samples than the original training Set(D).1: r=N/M # Calculate the class imbalance rate of the original dataset.2: b = random(r,1)3: a = int(M*b-N) *#* Calculate the number of samples to be expanded in Set(min).4: C = BIC(Set(min)) *#* Determine an optimal number of components.5: G_i_ = GMM(Set(min), C)  *#* Use GMM to cluster Set(min) into C clusters.6: **for** i ←1 to C **do**7:      Set(G″_i_) = {} # Create C empty sample sets.8:      **if**
$\left|\mathrm{Set}{(G^{\prime \prime }}_{i})\right|<a*(\left|G_{i}\right|/N)$ **then**
9:         $\mathrm{Set}(x\_\mathrm{new})=\mathrm{SMOTE}(G_{i},\mathrm{int}(a*(\left|G_{i}\right|/N)))$
       # Use SMOTE to synthesize new samples based on the G_i_ cluster samples.10:          G′_I_ = GMM(Set(min) + Set(x_new_k_), C)        # Use GMM again to cluster Set(min) and Set(x_new_k_) into C clusters.11:         **for** k *←*1 to int(a* num(G_i_)/N) **do**12:               **if** x_new_k_ in G′_i_ **then**              # The x_new_k_ is the k-th sample in Set(x_new).13:                  Add the x_new_k_ to Set(G″_i_)                    # Add the x_new_k_ sample to Set(G″i).14:               **if** x_new_k_ not in G′_i_ **then**15:                  Remove the x_new_k_
16:               **end if**17:               **end if**18:         **end for**19:      **end if**20:      Set(N′) = concatenate (Set(G″_i_))21: **end for**22: Set(D′) = concatenate (Set(max), Set(min), Set(N′))23: **return Set(D′)**

## 3. Wind Turbine Blade Bolt Fault Detection Model

**Figure 7:**
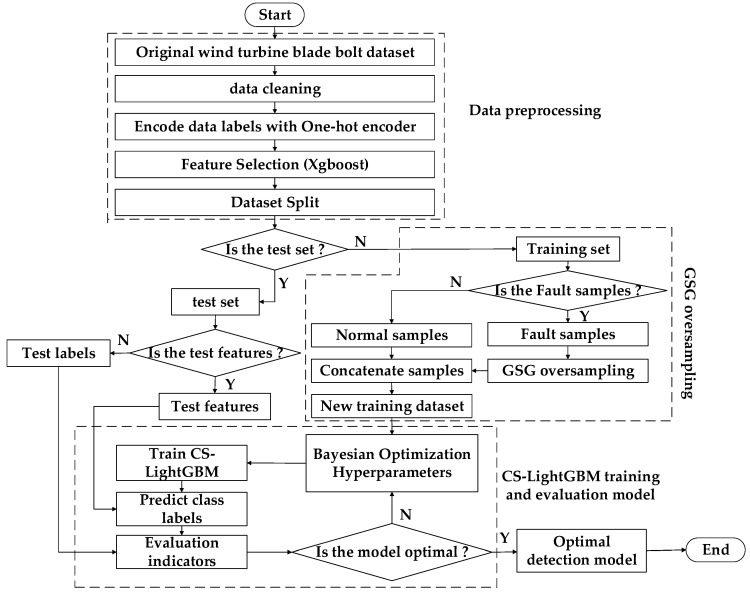
The fault detection model of Wind turbine blade bolts.

The fault detection model of wind turbine blade bolts is shown in Figure 7. It mainly consists of three main models: a data preprocessing model, a GSG oversampling model and a CS-LightGBM training and evaluation model.

### 3.1. Data Preprocessing

The data preprocessing model is mainly to perform relevant data processing operations on the original wind turbine blade bolts dataset, such as data cleaning and standardization, encoding the text labels of the dataset into numerical labels using one-hot encoder, and using XGboost to sort the importance of features for feature selection.

Data cleaning mainly processes the abnormal and missing values in the wind turbine blade bolts dataset. The dataset contains too many normal class samples; therefore, the method adopted in this paper is to directly remove the samples with abnormal or missing values in the normal class samples. The samples with abnormal or missing values in the fault class samples are filled with mean values, thereby reducing the loss of the fault class samples.

One-hot encoding mainly converts the text labels in the wind turbine blade bolts dataset into numerical labels for machine identification. Since its model is a binary classification problem, the normal labels and fault labels in the bolt operation data of wind turbine blades are defined as P = [0, 1].

When machine-learning algorithms learn data features, the best features are those that are sufficient and necessary. Fewer features can easily lead to the underfitting of the model, while too many features not only cause the overfitting of the model, but also take up too many computing resources. So, it is very important to select a certain number of features for the model. In this paper, the XGboost feature importance algorithm is used to rank the importance of the wind turbine blade bolts dataset features. The principle is to input each feature of wind turbine blade bolts data in a single decision tree. Then, calculate the degree value of the feature to the decision tree split point improvement performance measure. The degree value is weighted and counted by the leaf nodes responsible for weighting, and finally, according to the weighted value and count value to determine the importance of the feature. Its performance measurement is generally the Gini purity value of the selected split node. The dataset of wind turbine blade bolts in this study has a total of 48 features, and Table 1 lists the importance values of some features, where the initial letters of the feature parameters represent different blades of the same wind turbine. According to the feature importance value and expert experience, 11 features are selected as the feature training set.

Finally, the dataset after performing feature selection is data-standardized according to Equation (10) and its dataset is converted to a dataset with mean 0 and variance 1. This improves the training speed and accuracy of the wind turbine blade bolt fault detection model.

(10) $$x^{\prime }=\frac{x-\mu }{\delta }$$

where x′ is the standardized feature value, x is the original feature value, μ is the feature mean, and δ is the feature standard deviation.

### 3.2. GSG Oversampling

GSG is a new oversampling method proposed in this paper. GSG is mainly based on the fault class sample dataset in the wind turbine blade bolts training set, and expands the fault class samples to a certain number of scales when the distribution characteristics of the original fault class samples are kept unchanged as much as possible. Then, the expanded fault class sample dataset is concatenated into the normal class sample dataset to obtain a new training dataset. Thereby, the imbalance rate of normal class samples and fault class samples in the original training samples is alleviated.

### 3.3. CS-LightGBM Training and Evaluation Model

The CS-LightGBM training and evaluation model combines cost-sensitive learning with the LightGBM algorithm, which is used to train the new training set processed by GSG, and evaluate the classification performance of the model through the test set. Finally, through the Bayesian optimization algorithm, search the optimal hyperparameter combination, and obtain an optimal wind turbine blade bolt fault detection model.

#### 3.3.1. CS-LightGBM

Cost-Sensitive (CS), a new method in machine learning to process data classes’ imbalanced problems, is mainly used in the field of relevant classification algorithms. When the machine-learning algorithm classifies the samples, the cost of misclassifying one class of samples into another class of samples is very serious. Aiming at this problem, cost-sensitive learning assigns different costs to different misclassifications, so as to minimize the number of high-cost errors and the sum of the misclassification costs.

LightGBM is optimized on the basis of XGBoost, so its basic principle is basically similar to XGBoost; both use decision trees based on learning algorithms. LightGBM is mainly optimized for the training speed of the model, so the efficiency of LightGBM is generally higher than that of the XGboost algorithm. XGboost performs an indiscriminate split on the nodes of each layer, which wastes a lot of computing resources and time resources, as the information gain brought by some nodes to the algorithm model is almost 0. Aiming at the problem, LightGBM only selects the node with the largest splitting gain for splitting, and runs recursively under the depth limitation of the tree. In addition, LightGBM also performs parallel processing of data and data features, which speeds up the training speed, so that the overall performance of the LightGBM algorithm model is better than the XGboost algorithm model. Therefore, LightGBM is used as the classification algorithm of the detection model proposed in this paper.

CS-LightGBM, a method proposed in recent years to optimize the LightGBM classifier, introduces the idea of cost-sensitive learning into the LightGBM classifier. Specifically, it introduces a cost-sensitive function to replace the information gain in the weight function of the traditional LightGBM. So that when LightGBM performs iterative updates, the fault class samples can get excessive attention weights to improve the classification effect of class imbalance samples.

#### 3.3.2. Bayesian Optimization Algorithm

Bayesian Optimization (BO) is a global optimization algorithm based on probability distribution. When the hyperparameter search space of the algorithm model is given, BO constructs a probability model for the function to be optimized f: χ → Rd, and further uses the model to select the next evaluation point, and then iterates through the cycle to obtain the hyperparameter optimal solution:

(11) $$x*=\arg\min x_{x}{}_{\in \chi \subseteq R^{d}}f(x)$$

where x* is the optimal hyperparameter combination, χ is the decision space, and f(x) is the objective function.

The combination of different hyperparameters can affect the classification performance of a detection model, so it is very important to optimize the hyperparameters of the model. The LightGBM model has many hyperparameters, but some hyperparameters have little effect on the classification performance of the model. This study mainly optimizes four hyperparameters in the LightGBM model, as shown in Table 2.

#### 3.3.3. Model Evaluation Index

The performance evaluation index in this experiment is not only used the false alarm rate (FAR) and missing alarm rate (MAR) indexes to evaluate the classification performance of the fault detection model, but also introduces the F1-score index to better describe the classification performance of LightGBM classifier for unbalanced data.

The expressions of MAR, FAR, and F1-score are as follows:

(12) $$\mathrm{MAR}=\frac{\mathrm{FN}}{\mathrm{TP}+\mathrm{FN}}$$

(13) $$\mathrm{FAR}=\frac{\mathrm{FP}}{\mathrm{TN}+\mathrm{FP}}$$

(14) $$F1-\mathrm{score}=2\cdot \frac{P\cdot R}{P+R}$$

where P is the precision rate and R is the recall rate; and TP, TN, FP, and FN are the confusion matrices, as shown in Table 3.

## 4. Results

### 4.1. Sampling Effect Verification Experiment

To verify the effectiveness of the proposed sampling method in this paper, the GSG sampling method and traditional SMOTE sampling method were used to sample, respectively, the same simulated dataset. Then, the sampling effects based on the various methods were shown by data visualization. At the same time, in order to verify the sensitivity of the sampling method proposed in this paper to noise samples, after adding certain minority class noise samples to the simulation dataset, we then compared the effects of various sampling methods. In addition, to verify the sensitivity of GSG sampling method to the distribution characteristics of minority class data samples, some minority class sample sets with irregular distribution characteristics were randomly generated. The simulated simulation dataset consists of three clusters of sample sets obeying Gaussian distribution, whose specific distribution characteristics are shown in Table 4.

Here, the number of samples in the majority class accounts for 85%, and the samples in the minority class account for 15%. Dataset 1 is used as a majority class sample set, and dataset 2 and dataset 3 are combined to be used as a minority class sample set.

**Figure 8:**
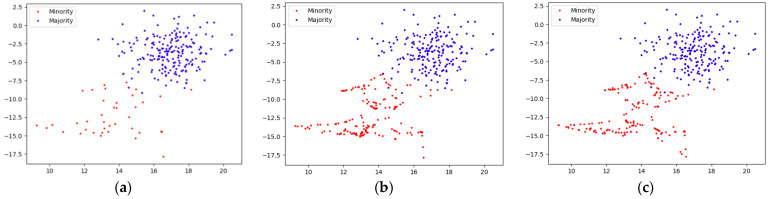
(**a**) Distribution of the original dataset that does not contain noisy samples; (**b**) Distribution of samples based on SMOTE sampling; (**c**) Distribution of samples based on GSG sampling.

Figure 8 shows the sampling effect based on different sampling methods in the same training set without adding noise samples, and the sampling strategy is 0.85. The original data distribution is shown in Figure 8a. Although there are no noise samples in the majority class samples, the majority class samples and the minority class samples still have certain overlapping samples in the boundary area. So as to examine the sampling situation of different sampling methods in the overlapping area.

It can be seen from Figure 8b,c that when the original training set does not contain noise samples. Both the traditional SMOTE and the GSG sampling method proposed in this paper have effectively sampled it, and the sample distribution characteristics after sampling roughly conforms to the distribution characteristics of the original samples. However, it is obvious that the traditional SMOTE sampling makes the boundary sample distribution tend to be uniform. In contrast, the samples obtained by the GSG sampling method have more distinct distribution characteristics within the minority class samples. In addition, the GSG sampling method also avoids the generation of overlapping samples as much as possible in the fuzzy area of the sample boundary.

**Figure 9:**
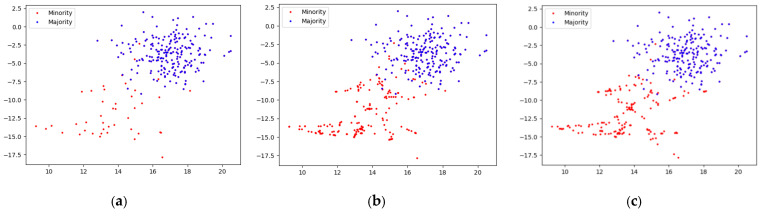
(**a**) Distribution of the original dataset containing the noisy samples; (**b**) Distribution of samples based on SMOTE sampling; (**c**) Distribution of samples based on GSG sampling.

Considering that the noise samples affect the sampling effect of various sampling methods, a certain number of minority class samples are added to the majority class samples as noise samples, and the original data situation is shown in Figure 9a. It can be seen from Figure 9b,c that when a certain a certain number of minority class noise samples are added to the majority class samples, the SMOTE will use the noise sample as exemplars to synthesize new samples. Therefore, the SMOTE sampling method synthesizes a large number of new samples between noise samples and between noise samples and normal samples. It makes the overlapping area more blurred and complicated, and, thus, the minority class samples lose their original distribution characteristics. However, the GSG sampling method first uses GMM to cluster the minority class samples, so that the noise samples are clustered into different clusters, and then synthesizes new samples in the intra-class, thus effectively reducing the situation of synthesizing new samples between the noise samples. In addition, when synthesizing new samples based on noise samples and normal samples, since the Euclidean distance between noise samples and normal samples is relatively far, the newly synthesized samples change the distribution characteristics of the original clusters. As a result, when the GMM is used to cluster data samples again, the probability of new synthetic samples being clustered into corresponding clusters is reduced and, thus, some synthetic samples are discarded. Therefore, the distribution characteristics of the original data samples can be retained to the greatest extent after the minority class samples are expanded.

To sum up, when there are no noise samples in the dataset, both traditional SMOTE and GSG can effectively sample the minority class samples, and retain the distribution characteristics of the original minority class samples to the greatest extent. However, the sampling effect of GSG is better than that of SMOTE in the case of intra-class distribution characteristics of minority class samples. When there are noise samples in the original dataset, traditional SMOTE is easily affected by the noise samples, making the overlapping area more blurred, and synthesizing new samples between the noise samples, thus changing the distribution characteristics of the minority class dataset. When GSG faces noise samples, it clusters the noise samples into different clusters, thereby reducing the situation of synthesizing new samples between noise samples and preserving the distribution characteristics of the original minority class samples to the greatest extent.

### 4.2. Wind Turbine Blade Bolt Fault Detection Experiment

#### 4.2.1. Wind Turbine Blade Bolts Data Description

In order to verify the effectiveness of the sampling method and fault detection model proposed in this paper, the operation monitoring data of a 2 MW wind turbine blade in a wind farm in Anhui Province were taken as the research object. The data source was mainly through the installation of fiber grating sensors and stress and other sensors at the blade root and hub of wind turbines for data collection, and the collection interval was 1 min. Some of the data are shown in Table 5. In order to avoid the randomness of the experiment, two datasets from different wind turbines were used in this experiment. The structure of each dataset is shown in Table 6.

#### 4.2.2. Experimental Results

To verify the effectiveness and superiority of the proposed method in this paper, two datasets were used as experimental objects in this experiment, as shown in Table 6. Based on each dataset, we compared the detection performance of the proposed fault detection model with CS-LightGBM, SMOTE-LightGBM, K-means-SMOTE-LightGBM, Borderline-SMOTE-LightGBM, SMOTE-CS-LightGBM, GSG-LightGBM models. In order to avoid randomness, each experiment was repeated 10 times, and then the average value was taken as the experimental result. Table 7 shows the experimental results.

As shown in Table 7, compared with other models, on the original dataset 1 and dataset 2. The new training set obtained by the sampling method proposed in this paper is input into the CS-LightGBM model, and the output FAR, MAR and F1-score value are all better than other models. For dataset 1, without considering the error term, we compared the fault detection model proposed in this paper with the traditional SMOTE-LightGBM fault detection model; the FAR is reduced by 2.39%, the MAR is reduced by 0.308%, and the F1-score is increased by 3.49%. For dataset 2, the FAR is reduced by 1.8%, the MAR is reduced by 0.576%, and the F1-score is increased by 2.67%.

Therefore, combined with the overall experimental results under the two datasets, the fault diagnosis model proposed in this paper has lower FAR, MAR and higher F1-score value than other models. Thus, the effectiveness and superiority of the method proposed in this paper are verified.

## 5. Conclusions

Wind turbine blade bolts operate under alternating loads for a long time, which often causes them to fail. When using machine-learning algorithms for fault detection, the fault detection performance is not only affected by external factors, but also affected by the data class imbalance. In order to solve this problem, this paper analyzed and compared the shortcomings of traditional sampling methods and algorithms, and proposed a fault detection method for wind turbine blade bolts based on GSG combined with CS-LightGBM. Its main contributions are as follows:

1. A new oversampling method GSG was proposed. GSG is based on the basic principle of GMM and SMOTE, which can retain the distribution characteristics of the original data to the greatest extent when the sample is expanded, avoiding the blindness of traditional SMOTE in performing oversampling. In addition, this method also effectively alleviated the influence of noise samples during oversampling, and effectively reduced the generation of overlapping samples.
2. We combined GSG and CS-LightGBM for the fault detection of wind turbine blade bolts. The model starts from expanding the fault class sample dataset and introducing cost-sensitive learning methods to solve the problem of data classes unbalanced in wind turbine blade bolts. Specifically, we used the GSG proposed in this paper to expand the fault class samples in the wind turbine blade bolts training dataset to obtain a new training dataset. Then, we inputted the new training dataset to the LightGBM classifier introducing a cost-sensitive function for training, and the operational status of the wind turbine blade bolt was used as the output value.
3. Both the proposed new sampling method and the fault diagnosis model were well experimentally verified. Analysis of the experimental results of GSG sampling on simulated datasets revealed that the sampling effect of GSG was better than that of traditional SMOTE, thus verifying the effectiveness of GSG. In addition, comparing the fault diagnosis model proposed in this paper with other models, the missing alarm rate and false alarm rate under the proposed model were lower, and the F1-score value was higher. Thus, the feasibility and superiority of the fault diagnosis model proposed in this paper were verified.

When the dataset has a serious class imbalance problem, the proposed fault detection model shows good detection performance. However, when there is only a slight class imbalance in the dataset, how to find an optimal sampling strategy for GSG needs further consideration, and will also be the focus of the next research.

## Figures and Tables

**Table 1:** Partial feature importance based on XGboost.

Feature	Importance	Feature	Importance	Feature	Importance
C-bolt-98.1°	0.254579	A-bolt-32.7°	0.060742	C-bolt-229.2°	0.013685
C-blade-180°	0.120046	A-blade-180°	0.023917	A-bolt-294.6°	0.009154
A-blade-90°	0.113605	A-bolt-196.5°	0.020874	B-bolt-294.6°	0.004120
...	...	...	...	...	...
B-bolt-32.7°	0.084388	A-bolt-0°	0.017649	C-blade-temp	0.0038916
B-bolt-98.1°	0.060742	C-bolt-98.1°	0.014236	C-blade-90°	0.000112

**Table 2:** Hyperparameters to be optimized for the LightGBM model.

Parameter	Range	Description
num_leaves	{1, 2, ···, n}	Number of leaves
max_depth	[3, 10]	Maximum tree depth
min_data_in_leaf	{1, 2, ···, n}	Minimum number of data in a leaf
learning_rate	(0, 1]	Learning rate

**Table 3:** Confusion matrix for classification results.

Actual Samples	Predicted Value	
	Predicted to Be a Failure Class Sample	Predicted to Be a Normal Class Sample
Failure class samples	TP	FN
Normal class samples	FP	TN

**Table 4:** Distribution characteristics of the simulation dataset.

Data Type	Dataset Name	Center Value	Variance Value	Total Number of Samples
Majority class dataset	1	[17, −4]	[2, 5]	226
Minority class dataset	2	[14, −10]	[3, 6]	20
	3	[13, −14]	[5, 0.4]	20

**Table 5:** Raw data of bolt part of wind turbine blades.

Feature Parameters	Time								
	03:27	03:28	03:29	03:30	03:31	03:32	…	07:24	07:25
A-bolt-0°	5.270	7.688	9.362	10.416	10.851	10.633	…	7.527	6.854
A-bolt-65.4°	8.835	9.207	9.393	9.362	9.176	8.990	…	7.967	7.471
A-blade-65.4°	−776	−772	−768	−752	−732	−720	…	−736	−752
…	…	…	…	…	…	…	…	…	…
C-blade-Temp	19.39	19.33	19.36	19.35	19.36	19.36	…	19.39	19.42

**Table 6:** The structure of the dataset.

Dataset Name	Total Number of Samples	Number of Normal Class Samples	Number of Fault Class Samples	Class Ratio
Dataset 1	4639	4282	357	12.994:1
Dataset 2	4203	3857	346	12.147:1

**Table 7:** Performance evaluation values based on different fault diagnosis models.

Dataset Name	Model	FAR (%)	MAR (%)	F1-Score
Data 1	CS-LightGBM	6.04 ± 0.024	0.467 ± 0.0029	0.959 ± 0.0025
	SMOTE-LightGBM	7.66 ± 0.028	0.682 ± 0.0041	0.945 ± 0.0040
	K-means-SMOTE-LightGBM	7.08 ± 0.022	0.605 ± 0.0038	0.950 ± 0.0029
	Borderline-SMOTE-LightGBM	6.84 ± 0.031	0.525 ± 0.0036	0.952 ± 0.0032
	SMOTE-CS-LightGBM	6.14 ± 0.019	0.465 ± 0.0022	0.956 ± 0.0027
	GSG-LightGBM	6.53 ± 0.023	0.528 ± 0.0046	0.953 ± 0.0036
	GSG-CS-LightGBM	**5.27 ± 0.016**	**0.374 ± 0.0023**	**0.978 ± 0.0012**
Data 2	CS-LightGBM	6.55 ± 0.030	0.471 ± 0.0024	0.957 ± 0.0028
	SMOTE-LightGBM	8.12 ± 0.026	1.002 ± 0.0025	0.938 ± 0.0024
	K-means-SMOTE-LightGBM	7.34 ± 0.029	0.773 ± 0.0029	0.946 ± 0.0027
	Borderline-SMOTE-LightGBM	6.96 ± 0.031	0.552 ± 0.0034	0.952 ± 0.0032
	SMOTE-CS-LightGBM	6.39 ± 0.024	0.507 ± 0.0021	0.958 ± 0.0019
	GSG-LightGBM	6.92 ± 0.028	0.499 ± 0.0027	0.954 ± 0.0026
	GSG-CS-LightGBM	**6.32 ± 0.021**	**0.426 ± 0.0018**	**0.963 ± 0.0021**

## Data Availability

The data that support the findings of this study are available from the corresponding author upon reasonable request.
